# Supplementary material for: Clinical presentation and comorbidities of obstructive sleep apnea-COPD overlap syndrome
Source: PLoS One. 2020 Jul 9;15(7):e0235331. doi: 10.1371/journal.pone.0235331 (PMC7347183; doi:10.1371/journal.pone.0235331)
Supplement: S1 Table — (DOCX) [file pone.0235331.s001.docx]

**E-table 1**: Clinical characteristics and cardiovascular comorbidities of patients without and with available spirometry data.

| **Clinical characteristics** | Items | Patients without spirometry data  n=30,320 | Patients with spirometry data  n=16,466 | *P* |
| --- | --- | --- | --- | --- |
| Age (years) |  | 58.2 [49.4; 66.7] | 57.7 [48.6; 66.1] | <0.01 |
| Gender (men) |  | 21268 (70.3) | 11693 (71.2) | 0.04 |
| Body mass index (kg/m2) |  | 30.5 [27; 35.1] | 31.1 [27.3; 35.9] | <0.01 |
| Smoking status | 0 | 18034 (59.7) | 8019 (48.8) | <0.01 |
| Smoking status | 1 | 7751 (25.7) | 5434 (33.1) |  |
| Smoking status | 2 | 4427 (14.7) | 2965 (18.1) |  |
| Alcohol |  | 1349 (4.5) | 816 (5) | 0.01 |
| Sedentarity |  | 4305 (14.2) | 3525 (21.5) | <0.01 |
| Apnea + hypopnea index (event/hour) |  | 33 [23; 48] | 34 [24; 50.6] | <0.01 |
| Oxygen desaturation index (event/hour) |  | 25 [15; 43] | 26 [14.1; 45.2] | 0.21 |
| Mean nocturnal Sa02 |  | 93 [91; 94] | 93 [91; 94] | <0.01 |
| pH |  | 7.4 [7.4; 7.5] | 7.4 [7.4; 7.5] | 0.32 |
| PaCO2 |  | 39 [36; 42] | 38 [35.8; 41] | <0.01 |
| PaCO2≥45 mmHg |  | 735 (12.2) | 649 (8.4) | <0.01 |
| PaO2 |  | 79 [72; 87] | 79 [71; 85.8] | <0.01 |
| **Cardiovascular comorbidities** |  |  |  |  |
| Hyperlipidemia |  | 8091 (26.9) | 4945 (30.4) | <0.01 |
| Hypertension |  | 13073 (43.4) | 7384 (45.1) | <0.01 |
| Peripheral arteriopathy |  | 551 (1.8) | 392 (2.4) | <0.01 |
| Coronary artery disease and myocardial infarction |  | 2288 (7.6) | 1344 (8.2) | 0.02 |
| Heart failure |  | 930 (3.1) | 400 (2.4) | <0.01 |
| Stroke |  | 1117 (3.7) | 494 (3) | <0.01 |
