# Supplementary material for: Clinical presentation and comorbidities of obstructive sleep apnea-COPD overlap syndrome
Source: PLoS One. 2020 Jul 9;15(7):e0235331. doi: 10.1371/journal.pone.0235331 (PMC7347183; doi:10.1371/journal.pone.0235331)
Supplement: Attachment — Submitted filename: Responses to reviewers PLoS one Overlap JLP.docx [file pone.0235331.s002.docx]

Manuscript number PONE-D-19-30703

Grenoble, 21 May 2020

Dear Dr Stelios Loukides, Academic Editor,

Please find enclosed the revised version of our article entitled **«Clinical presentation and cardiovascular outcomes of obstructive sleep apnea-COPD overlap syndrome»** by D. Adler et al., for consideration to be published in *PLOS ONE.*

Thank you for your e-mail regarding the above manuscript. We are grateful to the reviewers for their time and effort in evaluating our manuscript, and appreciate their constructive comments and criticisms. The following is a point-by-point response to the reviewers’ comments.

Yours sincerely,

Dr Dan Adler	Pr Jean-Louis Pépin

# Financial disclosure section:

*"This study was funded by an unrestricted grant from “Appel d’offre cohorte-fondation du souffle” (SRC_2017). SB, RT and JLP are supported by a research grant from the French National Research Agency (ANR-12-TECS-0010) in the framework of the “Investissements d’avenir” programme (ANR-15-IDEX-02) and the “e-health and integrated care” Chair of excellence of the University Grenoble Alpes Foundation. DA is supported by a research grant from the “Ligue Pulmonaire Suisse” to conduct a programme entitled “integrated care of patients surviving acute hypercapnic respiratory failure in the ICU”."*

*We note that one or more of the authors are employed by a commercial company: Private Practice Sleep and Respiratory Disease Centre.*

None of the authors was employed by a private company. Some of them (BS, YG, MS) exert in private medical practices but reimbursement of care went from French national public insurance. All the study funding was provided by academic grants.

# Answers to reviewer

We thank reviewers for the constructive comments and criticisms.

**Comments to the Author**

*Reviewer #1: This cross-sectional analysis of the French Sleep Apnoea Registry addresses an interesting question, namely how the co-existence of COPD and OSA (Overlap Syndrome) affects symptoms comorbidities compared to OSA alone.*

We thank the reviewer for her/his positive appreciation of our work.

*The sample size is large, however, the study design introduces a lot of bias that hampers the interpretation of the findings. In addition, statistically significant differences in symptom burden are reported between Overlap Syndrome and COPD alone. However, when looking at the difference and the confidence intervals, there is no clinically relevant difference between the groups. Therefore, the conclusion is not really supported by the data.
The question asked and the data are interesting but the title, the methods (hypothesis, outcomes), the interpretation of the data, the limitations and the conclusions need to be revised thoroughly.*

The following is point by point response to reviewer concerns and comments.

*- Mean (SD) or median (IQR) AHI, ODI and SpO2 should be reported in the abstract to understand differences in SDB in the two compared populations*

We agree and thank for this suggestion. The following sentence has been included in the abstract:

“Apnea hypopnea index (36 [25; 52] vs 33.1 [23.3; 50]), oxygen desaturation index (28 [15; 48] vs 25.2 [14; 45]) and mean nocturnal SaO2 (92 [90; 93.8] vs 93 [91.3; 94]) were significantly more altered in the OVS group.”

*- The method section of the abstract should report outcomes of interest and statistical approach*

This has been done by adding the following sentence:

“Univariable and multivariable logistic regression models were used to compare OVS versus OSA alone on symptoms and cardiovascular health.”

It was difficult to add more details as space is limited in the abstract.
*- the difference in ESS between the compared populations is statistically significant but there is no clinically meaningful difference (MCID in ESS 2-3), therefore, the conclusion is not supported* *by the data*

We agree that the ESS difference is below the MCID. We have tone down the conclusion sentences in the abstract and in the main text and included the following sentence and references in the discussion:

“This difference regarding sleepiness is statistically significant but under the minimum Clinically Important Difference of -2 or -3 [36,37].”

*- patients with OSA who are in the French register represent a population referred to a sleep clinic due to suspicion of symptomatic SDB whereas patients with Overlap Syndrome might have undergone a sleep study for a different reason; this introduces a relevant selection bias that hampers the interpretation on differences in sleepiness and associated symptoms*

We would like to respectfully say that the conditions of referral are the same for the two populations in the French registry. Our study has limitation but we do not think that this one is significant.

*- due to the study design, these results can be used for generating a hypothesis but not more*

We agree with the reviewer and we have modified the following sentence in the study limitation paragraph:

“In such a well-maintained registry, a careful analysis of confounders allowed reporting an association between COPD and cardiovascular health in OSA patients [44] but causality remains to be firmly established by incident data and randomized controlled trials as our study design only allow generating hypothesis.”

*- the title should be changed to symptoms and comorbidities – delete cardiovascular outcomes); this is a cross-sectional analysis not a longitudinal analysis that looks at how baseline characteristics affect the incidence of cardiovascular events*

This has been done. Thanks, we agree that the title is more accurately reflecting the data now.

*- it should be discussed which scoring rules have been used during the period analysed (1997-2017); hypopnea definition has changed and this will affect the prevalence of Overlap Syndrome*

“The majority of centers were using a 3% desaturation and a micro arousal ending the event for hypopneas scoring during polysomnography and reduced flow associated with a 3% desaturation for hypopnea scoring during type III sleep recording. Owing to the size of the registry it was impossible to use a centralized scoring for all sleep studies but we did not observe major differences in AHI between polysomnography and type III sleep recording (not reported)”

This paragraph has been included in the limitation section of the discussion.

*Reviewer #2: This cross-sectional study evaluates and compares the clinical characteristics of patients diagnosed with OVS and moderate OSA alone, in the largest until now study, retrieved from the French National Sleep Apnea Registry. In addition, emphasis was placed on the impact that COPD may have on cardiovascular outcomes in patients with OSA. COPD diagnosis was based on available spirometric data, while OSA was diagnosed using either PSG or type III cardio-respiratory studies. The percentage of OVS patients out of the total examined OSA population was in accordance with published epidemiological studies on this field (at 13%), whereas OSA related symptoms were less frequently reported in the OVS than the OSA population (apart from nocturia). Finally, and more importantly, increasing COPD severity, based on GOLD airflow limitation criteria, associated with higher prevalence of CVD, but not with hypertension and stroke after adjustment for significant cofounders.*

*Major concerns*

*1. It would be interesting to distinguish between patients diagnosed with PSG or type III studies and to perform the same analyses. The findings of this proposed subgroup analysis may explain possible differences regarding the impact of COPD on prevalent hypertension as well as other important findings such as symptoms related to OSA between investigated groups.*

We fully agree with the reviewer and thank for this comment. The tables below are depicting the results in the respective population of patients being diagnosed by polygraphy or polysomnography. You will see that the trends are the same and the message is unchanged. There is only a reduction in power in this subgroup analysis. We propose not to include these tables but add the following sentence in the results section:

“A subgroup analysis in patients diagnosed by respiratory polygraphy or polysomnography yielded to similar results.”

*2. The authors should consider the fact that hypercapnic OSA patients (6.7% of the patients in this study) may have obesity hypoventilation syndrome, especially since the reported median BMI is 31.1 kg/m2. FVC values could also be helpful in this. I would suggest adding this, at least as a limitation because those patients may not represent a “pure” OSA population.*

We thank the reviewer for this comment. We have included the following sentence in the discussion section:

“A significant percentage of OSA patients with hypercapnia were obese representing an obesity hypoventilation population included in the general OSA population.”

*3. It would be also useful to better define COPD, underling the fact that included patients should be ≥40 years and have a history and symptoms compatible with a COPD diagnosis. How the authors explain that 33.7% of the OVS patients were non-smokers?*

One of the strength of the registry is that COPD diagnosis was not self-reported by patients but validated by respiratory physicians. We agree that a prevalence of more than 33% of non-smokers is more than intuitively expected. However, exiting available data estimated that 25-45% of patients with COPD have never smoked [Salvi 2009 and Tan 2015].

***Proportion of patients with COPD who are non-smokers worldwide***

(From Salvi SS; Lancet 2009; 374: 733-743)

*4. The authors should also add minimum SaO2 values dying sleep in Table 1. They should also remove peripheral arteriopathy from Table1 or to add other comorbidities.*

We have removed “peripheral arteriopathy”from table 1. Data of minimal nocturnal SaO2 are unfortunately not available in the registry.

*5. In the present study, OVS was not associated with prevalent stroke. Were there any available data regarding the subtypes of stroke in this study (i.e. ischaemic or haemorragic)? Actually, COPD may especially increase the risk of hemorrhagic stroke, while the risk of ischemic stroke in COPD patients could be confounded by other parameters. Similarly, non- significant effect of COPD on prevalent hypertension may be hard to explain. Nevertheless, moderate to severe and very severe COPD are all associated with multimorbidity, making it difficult to find any difference in prevalent hypertension.*

Unfortunately we have no detailed information on the subtypes of stroke in this large registry. This has been added as a limitation of the study.

*Minor concerns*

*1. The authors state that “Randomized controlled trials comparing clinical outcomes among OVS patients with OSA treated with CPAP versus OVS patients with untreated OSA are highly* *desirable.”
Although RCTs are the cornerstone of medical evidence and explain causality, they are hard to be implemented in daily practice due to ethics concerns regarding treatment of OSA. Thus, real life studies, as the present one, could offer important evidence on the field of OVS.*

We agree and we thank the reviewer for this comment.

*2. Discussion section: the authors state that “Sustained hypoxemia may also adequately assess the degree of sympathetic activity.” They should provide more clarifications and references.*

Sorry if our sentence was unclear. The sentence has been suppressed and replaced by the following paragraph and a reference in the discussion to clarify the point:

“Diurnal and nocturnal hypoxemia of COPD is also associated with elevated sympathetic nerve activity, which is probably linked to an increased cardiovascular risk [40].”
